# The major TMEM106B dementia risk allele affects TMEM106B protein levels and myelin lipid homeostasis in the ageing human hippocampus

**DOI:** 10.21203/rs.3.rs-2392941/v1

**Published:** 2023-01-17

**Authors:** Jun Yup Lee, Dylan Harney, John Kwok, Mark Larance, Anthony Simon Don

**Affiliations:** The University of Sydney SMS: The University of Sydney School of Medical Sciences; The University of Sydney; The University of Sydney SMS: The University of Sydney School of Medical Sciences; The University of Sydney SMS: The University of Sydney School of Medical Sciences; The University of Sydney School of Medical Sciences

**Keywords:** TMEM106B, proteomic, lipidomic, sphingolipid, neurodegeneration, ageing, myelin, hippocampus

## Abstract

**Background:**

The risk for dementia increases exponentially from the seventh decade of life. Identifying and understanding the biochemical changes that sensitize the ageing brain to neurodegeneration will provide new opportunities for dementia prevention and treatment. This study aimed to determine how ageing and major genetic risk factors for dementia affect the hippocampal proteome and lipidome of neurologically-normal humans over the age of 65. The hippocampus was chosen as it is highly susceptible to atrophy with ageing and in several neurodegenerative diseases.

**Methods:**

Mass spectrometry-based proteomic and lipidomic analysis of CA1 hippocampus samples from 74 neurologically normal human donors, aged 66–104, was used in combination with multiple regression models and gene set enrichment analysis to identify age-dependent changes in the proteome and lipidome. ANOVA was used to test the effect of major dementia risk alleles in the *TMEM106B* and *APOE* genes on the hippocampal proteome and lipidome, adjusting for age, gender, and post-mortem interval.

**Results:**

Forty proteins were associated with age at false discovery rate-corrected P < 0.05, including proteins that regulate cell adhesion, the cytoskeleton, amino acid and lipid metabolism, and ribosomal subunits. Transmembrane protein 106B (TMEM106B), a regulator of lysosomal and oligodendrocyte function, was regulated with greatest effect size. The increase in *TMEM106B* levels with age was specific to carriers of the rs1990622-A allele in the TMEM106B gene that is associated with increased risk for frontotemporal dementia, Alzheimer’s disease, Parkinson’s disease, and hippocampal sclerosis with ageing. Hippocampal lipids were not significantly affected by *APOE* genotype, however levels of myelin-enriched sulfatides and hexosylceramides were significantly lower, and polyunsaturated phospholipids were higher, in rs1990622-A carriers after controlling for *APOE* genotype.

**Conclusions:**

Our study provides the first evidence that TMEM106B protein abundance is increased with brain ageing in humans, and the first evidence that the major *TMEM106B* dementia risk allele affects brain lipid homeostasis, with a clear effect on myelin lipid content. Our data implies that *TMEM106B* is one of a growing list of major dementia risk genes that affect glial lipid metabolism.

## Background

Ageing is the dominant, unifying risk factor for all major forms of dementia, and is thought to constitute the prodromal phase of neurodegeneration. An estimated 60–80% of dementia cases are Alzheimer’s disease (AD) [1], in which the hippocampus, a region of the brain that is essential for learning and memory, is affected early and heavily by neurofibrillary tangle (NFT) pathology and atrophy [2, 3]. Hippocampal pathology and degeneration are also characteristic of hippocampal sclerosis with ageing (HS-ageing), limbic-predominant age-related TDP-43 encephalopathy (LATE), frontotemporal dementia (FTD) and dementia with Lewy bodies (DLB) [4–7]. Further to these dementia-related pathological changes, the hippocampus loses volume in the course of normal ageing [8], and this is associated with reduced verbal memory performance [3, 9].

Proteomic mass spectrometry permits the unbiased identification of cellular and biochemical changes in the brain as a function of ageing. This includes changes that cannot be detected using transcriptomics, such as molecular signatures arising from defective turnover and clearance pathways implicated in brain senescence [10, 11]. While there have been many studies characterizing transcriptomic changes in the ageing human brain [12, 13], few have investigated proteomic changes. Proteomic analyses of the rat, monkey, and human hippocampus reported changes to electron transport proteins [14–17], lysosomal proteins [14], redox control [15, 16], RNA splicing [14], and ribosomal stoichiometry [15] with increased age. However, the existing proteomic datasets for normal ageing in the human hippocampus are limited by small sample sizes [14, 17]. A recent, larger proteomic analysis of human frontal cortex demonstrated downregulation of mitochondrial and synaptic proteins in AD cases, whereas only synaptic proteins were inversely correlated with physiological ageing (ages 30 to 69) [18]. Another study established that mitochondrial, synaptic, and inflammatory protein networks are associated with cognitive trajectory [19], but did not investigate the effects of ageing on the brain proteome. Although lipids make up > 50% of the dry weight of the brain [20], there have been very few studies on changes to the brain lipidome with ageing [21, 22].

Dementia risk doubles every 5 years after age 65 [1]. We therefore aimed to identify changes in the hippocampal proteome and lipidome of neurologically-normal humans over the age of 65 that sensitize to neurodegeneration. Protein networks associated with cytokine signaling and axon guidance were reduced with hippocampal age, whereas ribosomal, amino acid metabolism, and oxidative phosphorylation networks were increased. Levels of the lysosomal protein transmembrane protein 106B (TMEM106B) showed the greatest increase with ageing, and this effect was driven by the rs1990622-A allele in the *TMEM106B* gene, which is associated with cognitive decline in ageing and significantly increased risk for multiple forms of dementia. No lipids were significantly associated with age at death, however the *TMEM106B* rs1990622-A allele was associated with reduced myelin sphingolipid and higher polyunsaturated phospholipid content in the hippocampus. This is the first study to show an effect of this major dementia risk allele on brain lipid homeostasis.

## Methods

### Human tissue samples

Fresh-frozen hippocampus tissue samples (CA1 region) were provided by the New South Wales Brain Tissue Resource Centre (NSW BTRC) and Queensland Brain Bank. Samples were from donors with no neurological disorders at the time of death. Braak staging was performed as described [23], and showed an absence of NFT pathology (Braak 0) or NFT pathology restricted to the entorhinal cortex (Braak I/II) in the 66 cases for which this information was available [2]. Braak stage was not available for 8 donors. Age, post-mortem interval (PMI), *APOE* genotype, gender, *TMEM106B* rs1990622 genotype, and Braak stage for each sample is provided in the Additional File 1. These cases were used for a previous targeted lipid analysis [24], however this paper reports new proteomic and lipidomic results and analyses. This work was approved by the University of Sydney Human Research Ethics Committee, approval #HREC2016/801.

Homogenates were prepared by placing 10–20 mg of frozen tissue into 500 mL of 20 mM Hepes pH 7.4, 10 mM KCl, EDTA-free cOmplete protease inhibitor cocktail (Roche), 1 mM dithiothreitol, and 3 mM β-glycerophosphate, and ultrasonicating for 5 min (30s on/30s off) at 4°C in a Qsonica Q800R2 sonicating bath. Homogenates were cleared by centrifugation at 1000·g for 10 min at 4°C, and the supernatant was stored at −80°C in 100 μL aliquots. Total protein concentrations were determined using the Bradford assay (Bio-Rad).

### Proteomic sample preparation

Crude homogenate (10 μg protein) was extracted with 100 μL 4% SDS, 100 mM NaCl, 20 mM NaPO_4_ (pH 6), 10 mM NaF, 10 mM tris(2-carboxyethyl)phosphine (TCEP), 10 mM N-ethylmaleimide (NEM), 10 mM sodium pyrophosphate, 2 mM sodium orthovanadate, 60 mM sodium β-glycerophosphate, and EDTA-free cOmplete protease inhibitor cocktail (Roche). The volume was made up to 150 μL using MilliQ water and samples were incubated for 10 min at 65°C with shaking (500 rpm), then ultrasonicated for 10 min at 20°C (15s on/15s off). Proteins were precipitated with chloroform/methanol/water in the ratio 1:4:1:3 (sample:methanol:chloroform:water) [25], dried down, and reconstituted in 30 μL of 8 M Urea in 0.1 M Tris-HCl (pH 8.0). Protein concentrations were measured by BCA assay (ThermoFisher Scientific), after which protein samples were diluted 8-fold in 0.1 M Tris-HCl (pH 8)/1 mM CaCl_2_ and digested for 16 h at 37°C with 0.1 μg trypsin (#90058, ThermoFisher). Digestion was stopped with the addition of trifluoroacetic acid to a final concentration of 1%, and the samples were centrifuged to pellet any undigested protein (18,000×g, 10 min, 20°C). The supernatants were transferred to new tubes and subjected to solid-phase extraction [26].

### Proteomic analysis by nano-flow liquid chromatography-tandem mass spectrometry (LC-MS/MS)

Quantitative proteomics was conducted using data-independent acquisition on a Thermo Scientific Q-Exactive HF-X mass spectrometer coupled to an EASY-nLC system, using previously described settings [27]. Retention time-normalised spectral libraries were used for targeted data extraction using Spectronaut [27], yielding normalised abundances for 2615 proteins. Proteins that were detected in three or fewer samples were excluded from subsequent analyses, leaving 2091 proteins. Two samples were removed due to the absence of quality protein signals (< 800 proteins identified).

### Linkage disequilibrium analysis for rs1990622 single-nucleotide polymorphism (SNP)

Linkage disequilibrium between rs1990622 and all SNPs located ± 500,000 bp from the *TMEM106B* gene was determined using the R package LDlinkR using the LDproxy function [28]. The squared correlation coefficients (r^2^) between rs1990622 and other SNPs was used as the measure of linkage strength, where r^2^ = 1 represents perfect linkage disequilibrium (coinheritance of SNPs). Analysis was performed using the 1000 Genomes Project database for Utah residents with Northern and Western European ancestry (CEU) and British in England and Scotland (GBR) populations [29].

### DNA extraction and TMEM106B rs1990622 genotyping

DNA was extracted from brain tissue using phenol-chloroform extraction [30]. Genotyping was performed using a TaqMan SNP genotyping assay for *TMEM106B* rs1990622 (ThermoFisher, Assay ID C_11171598_20, Catalog #4351379) as per manufacturer instructions. *APOE* genotypes for these cases were reported previously [24].

### Lipidomic analysis by LC-MS/MS

Lipids were extracted from hippocampal homogenates (~ 100 μg protein) using a one phase butanol-methanol (BUME) (1:1 v/v) procedure [31]. The following internal standards were added to each sample: 5 nmoles of d19:0/19:0 PC, 2 nmoles of d18:1/17:0 SM, d18:1/12:0 HexCer, 17:0/17:0 PS, 17:0/17:0 PE, 17:0/17:0/17:0 TG, 17:0/17:0 PG, 14:0/14:0/14:0/14:0 cardiolipin, 17:0 cholesteryl ester, 1 nmole of 17:0/17:0 PA, d18:1/15:0-d7 PI, cholesterol-d7, 0.5 nmoles of d18:1/17:0 ST, d18:1/17:0 ceramide, 17:1 LPE, 17:1 LPS, 17:0 LPC, 18:1-d7 monoacylglycerol, d18:1/15:0-d7 diacylglycerol, d18:1/12:0 Hex2Cer, and 0.2 nmoles of 17:1 sphingosine, 17:1 sphingosine 1-phosphate, d3-16:0 acylcarnitine, 17:0 LPA.

Lipids were detected using multiple reaction monitoring on a TSQ Altis triple quadrupole mass spectrometer with Vantage HPLC (ThermoFisher Scientific). Precursor and product ion pairs are listed in Additional File 1. Lipids were resolved on a Waters Acquity UPLC CSH 2.1 × 100 mm C18 column (1.7 μm particle size) at a flow rate of 0.28 ml/min. Mobile phases were A: 10 mM ammonium formate, 0.1% formic acid, 60% acetonitrile and 40% water; B: 10 mM ammonium formate, 0.1% formic acid, 10% acetonitrile and 90% isopropanol. Run time was 25 min using a binary gradient starting at 20% B for 3 min, increasing to 45% B from 3–5.5 min, then to 65% B from 5.5–8 min, then to 85% B from 8–13 min, then to 100% B from 13–14 min. The gradient was held at 100% B from 14–20 min, then decreased to 20% B and held to 25 min. TraceFinder 4.1 (ThermoFisher) was used to integrate the peaks. The molar amount of each lipid was calculated with reference to its class-specific internal standard. As per Lipidomics Standards Initiative guidelines, each lipid was expressed as a molar % of total lipid quantified in that sample.

### Data analysis

All data and statistical analyses were performed using R. An a priori multiple regression power calculation was performed using the R package ‘pwr’ (pwr.f2.test) with 3 variables, a coefficient of determination (R^2^) of 0.3, Bonferonni corrected significance level under the assumption that 2000 proteins are quantified (α = 0.05/2000), and power set at 0.8, giving a conservative optimal sample size of 84.

Unless otherwise indicated, P values were adjusted for multiple comparisons using the Benjamini-Hochberg false discovery rate (FDR) correction at 5% (i.e. Q < 0.05 was considered significant). The normal distribution of residuals for all statistical models was assessed using the Anderson-Darling normality test. In cases where the test indicated non-normally distributed residuals, QQ plots and histograms were used to assess the degree of deviation from normality to decide whether variables should be natural log-transformed to fulfil the assumptions of normal distribution. Models that produced significant results but not fulfilling the assumptions of normal distribution with both linear and natural log-transformed continuous variables were considered false positives. One sample was identified as an outlier in both the proteomic and lipidomic datasets by hierarchical clustering and dendrogram analysis, using the hclust() function in R with Euclidean distance measures between all samples. This sample was excluded from further analyses.

Associations between age and protein levels were tested by multiple regression with PMI and sex as covariates (numerical variables natural log-transformed). The interaction between rs1990622 genotype and age in affecting TMEM106B protein levels was assessed using a multiple regression model, adjusting for PMI and sex. One-way ANOVA was used to test the effect of rs1990622 on TMEM106B protein levels, adjusting for age, PMI, and sex. Multiple regression models adjusted for age, PMI, and sex were used to test correlations between TMEM106B protein levels and the other 2090 proteins in the data set. Effects of *TMEM106B* rs1990622 and *APOE* genotype on the hippocampal lipidome and proteome were tested using one-way ANOVA, adjusting for age, PMI, and sex.

### Gene set enrichment analysis

Gene set enrichment analysis (GSEA) was performed using the clusterProfiler package [32]. The t-statistic value from each of the 2091 linear models assessing the association between age and the levels of each protein were used to rank proteins from the most positively to the most negatively correlated with age. The GSEA algorithm was then applied to this list using the GSEA() function, which checks whether a predefined set of proteins (e.g. mitochondrial oxidative phosphorylation) clusters at the top or bottom of the list to detect any coordinated upregulation or downregulation of protein sets with age. Protein sets used for the analyses were obtained from gene sets in the Molecular Signatures Database (v7.5.1 MsigDB, released January 2022). Gene sets containing less than 15 or more than 200 genes were excluded from the analyses. Statistical significance was determined based on permutation testing (10,000 permutations) using randomly generated gene lists, and significant enrichment was determined on the basis of Benjamini-Hochberg’s false discovery rate corrected P value of 0.05 (Q < 0.05). This workflow was also used for detecting coordinated changes in the proteome that are associated with TMEM106B protein abundance.

## Results

Ribosomal and respiratory proteins increase, and axon guidance proteins decrease, with age in the human hippocampus

**Figure 1:**
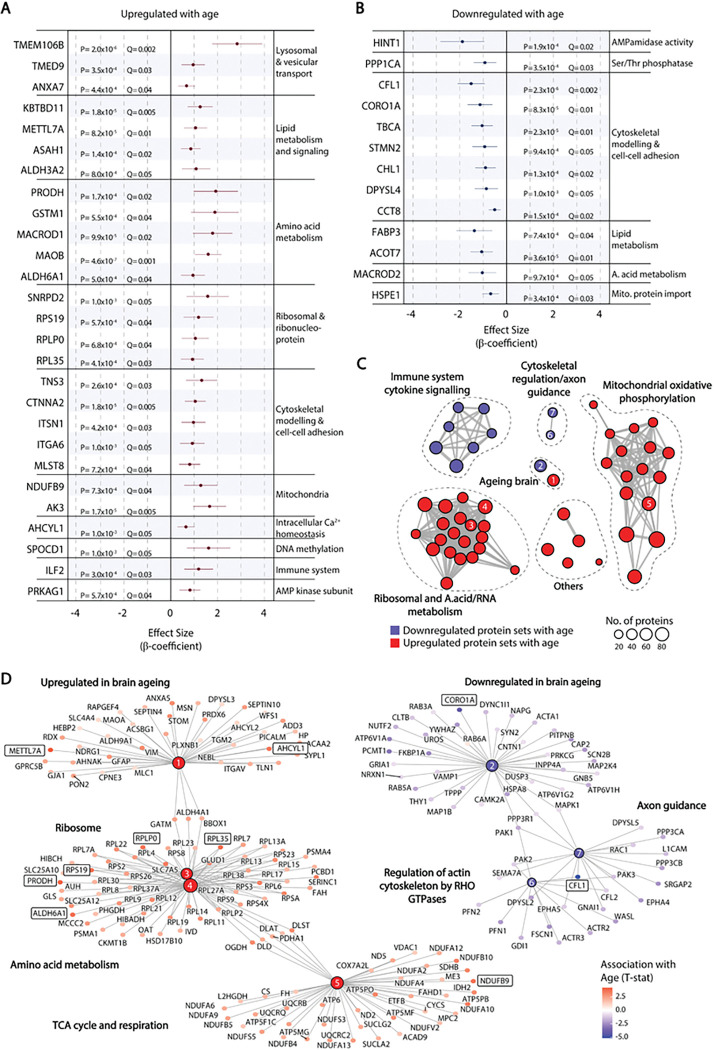
Proteins significantly correlated with age in human hippocampus. (A-B) Effect size estimates and 95% confidence intervals (horizontal bar) for proteins positively (A) or negatively (B) correlated with age at death (Q<0.05) in linear regression models adjusted for PMI and sex. (C) Protein set enrichment map of all significantly enriched categories from GSEA using curated protein sets from the molecular signatures database (MSIGDB, C2). Each node represents a significantly enriched protein set at Q<0.05 that is downregulated (blue) or upregulated (red) with age. Protein sets with overlapping membership are connected by edges, where the thickness indicates the number of overlapping proteins. (D) Protein membership of specific nodes shown in (C). Nodes have been numbered to identify their position in the protein set enrichment map (C). Individual proteins that were significantly correlated with age at Q<0.05 (A,B) are boxed.

To identify proteins whose levels are regulated by age in the human hippocampus, proteomic analysis was performed on frozen tissue samples from the hippocampus CA1 region of 74 neurologically normal donors aged 66–104 (mean age at death 78 ± 8.4 years, 61% male). Braak staging for NFT pathology was available for 66 samples, and all of these were Braak stage 0-II, indicating the absence of hippocampal NFT pathology that precedes neurodegeneration in AD [2, 23]. After adjusting for false discovery rate (Q < 0.05) 27 proteins were positively correlated (Fig. 1A) and 13 negatively correlated (Fig. 1B) with age at death. These included components of ribosomes and mitochondria, as well as proteins involved in lysosome transport, lipid metabolism, amino acid metabolism, cytoskeletal modelling and cell-cell adhesion. Lysosomal transmembrane protein TMEM106B was regulated with the greatest effect size, increasing with age at death.

Gene set enrichment analysis (GSEA) was applied to identify coordinated changes affecting biologically related groups of proteins. Proteins positively and negatively correlated with age in our dataset were significantly enriched in 42 and 10 curated gene sets in the Molecular Signatures Database (v7.5.1 MsigDB), respectively (Supplementary Table 1). Protein networks related to cytokine signaling, cytoskeletal regulation and axon guidance were decreased with hippocampal ageing, while protein networks involved in protein translation (ribosomes), amino acid and nucleotide metabolism, and oxidative phosphorylation were increased (Fig. 1C and D). Proteins that were positively associated with ageing in our dataset were significantly enriched for those whose gene expression increases with brain ageing, and downregulated proteins were enriched for those whose gene expression decreases with brain ageing in the human frontal cortex [13] (Fig. 1C and D). Importantly, several proteins that were significantly correlated with age at Q < 0.05 (Fig. 1A and B) were members of these significantly enriched protein sets (Fig. 1D). TMEM106B was not a member of any protein sets that were significantly enriched in GSEA, suggesting that its upregulation does not form part of a coordinated response to ageing that can be categorized by predetermined gene sets.

### The correlation between TMEM106B and age is driven by the rs1990622-A dementia risk allele

**Figure 2:**
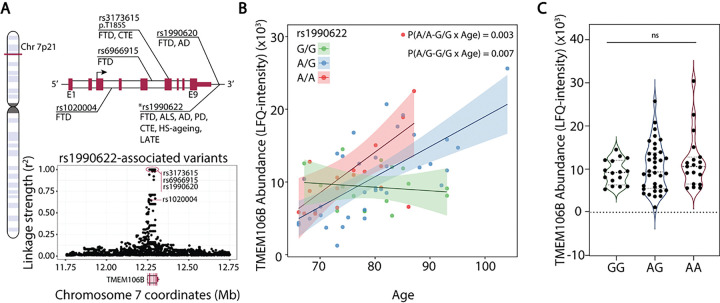
Hippocampal TMEM106B levels increase in carriers of the rs1990622-A risk allele. (A) (Top) Gene map of the *TMEM106B* locus (chromosome 7p21) with annotations of key *TMEM106B* SNP loci associated with neurodegenerative diseases (FTD: frontotemporal dementia; ALS: amyotrophic lateral sclerosis; AD: Alzheimer’s disease; PD: Parkinson’s disease; CTE: chronic traumatic encephalopathy; HS-ageing: hippocampal sclerosis with ageing; LATE: limbic-predominant age-related TDP-43 encephalopathy). Exons (E1-E9) are denoted in red, introns in white, and non-coding regions as a line. (Bottom) Linkage disequilibrium between rs1990622 and all SNPs located ±500,000 bp from the *TMEM106B* gene as measured by the squared correlation coefficient (r^2^, where r^2^ = 1 is perfect linkage, depicted in red oval). Dementia risk-associated SNPs depicted in the gene map are labelled on the linkage plot. (B) TMEM106B protein levels as a function of age and rs1990622 genotype. P values refer to the interaction between rs1990622 genotype and age in multiple regression adjusted for PMI and sex. (C) TMEM106B levels as a function of rs1990622 genotype after adjusting for age, PMI, and sex (ANOVA, F = 2.7, P = 0.07).

Several recent studies have reported that TMEM106B forms amyloid fibrils with ageing and in neurodegenerative diseases [33–35]. The precise biochemical function of TMEM106B is unknown, however a dominantly inherited amino acid substitution (D252N) in the protein causes hypomyelinating leukodystrophy [36] and myelination defects are observed in TMEM106B knockout mice [37, 38]. At the cellular level, TMEM106B regulates lysosomal pH, size, transport and positioning [37–40]. Additionally, a SNP in the 3’ untranslated region of *TMEM106B* (rs1990622-A) is associated with increased risk for AD, FTD, Parkinson’s disease, HS-ageing, and LATE [4, 39, 41, 42], and is in perfect linkage disequilibrium with other SNPs in the *TMEM106B* gene that modify dementia risk (Fig. 2A). We obtained rs1990622 genotype frequencies of 49% G and 51% A (26% A/A, 51% A/G, 23% G/G) for our cohort, in agreement with reported values [42]. The increase in hippocampal TMEM106B levels with age was specific to carriers of the rs1990622-A risk allele and not observed in homozygous carriers of the protective G allele (Fig. 2B). Rs1990622 did not affect TMEM106B protein levels after adjusting for age (Fig. 2C).

### TMEM106B levels are associated with lipid metabolic and myelination proteins

**Figure 3:**
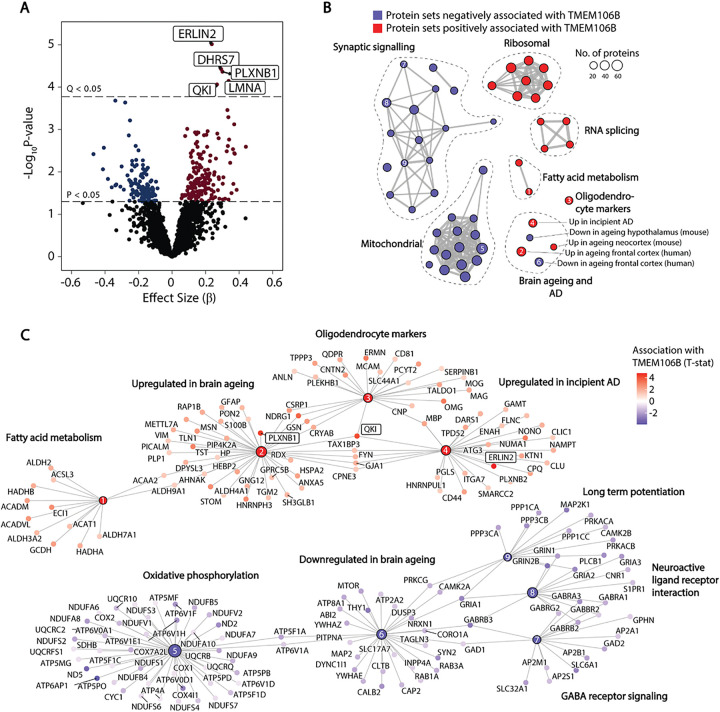
TMEM106B levels are correlated with proteins involved in myelination and brain ageing. (A) Proteins correlated with TMEM106B in multiple regression adjusted for PMI, sex, and age. Proteins significant at Q<0.05 are boxed and labelled. (B) Protein set enrichment map of selected significantly enriched categories from GSEA using curated protein sets from the molecular signatures database (MSIGDB, C2). Each node represents a significantly enriched protein set at Q<0.05 that is negatively (blue) or positively (red) associated with TMEM106B levels. Protein sets with overlapping membership are connected by edges, where the thickness indicates the number of overlapping proteins. (C) Protein membership of specific nodes shown in (B). Nodes have been numbered to identify their position in the protein set enrichment map (B). Proteins that were significantly correlated with TMEM106B levels at Q<0.05 are boxed.

To gain further insight into the function of TMEM106B and the implications of higher TMEM106B levels in the hippocampus, we identified proteins whose levels were correlated with TMEM106B after adjusting for age (Fig. 3A). Five proteins were positively correlated with TMEM106B at Q < 0.05: ER lipid raft associated 2 (ERLIN2), an endoplasmic reticulum protein that regulates lipid metabolism [43] and cell cycle progression [44]; dehydrogenase/reductase 7 (DHRS7), a member of the short-chain dehydrogenase/reductase family involved in the metabolism of retinoids and sterols [45]; the RNA-binding protein quaking I (QKI), a master regulator of oligodendrocyte differentiation, lipid biosynthesis, and myelination [46, 47]; the semaphorin receptor plexin B1 (PLXNB1), which is involved in synapse formation and axonal guidance [48, 49]; and nuclear envelope protein lamin A/C (LMNA). No proteins were regulated by rs1990622 genotype at Q < 0.05 (Supplementary Table 2).

GSEA demonstrated that TMEM106B levels are negatively associated with synaptic signaling and oxidative phosphorylation protein networks, and positively associated with ribosomal, RNA splicing, fatty acid metabolism, and oligodendrocyte protein networks (Fig. 3B and C, and Supplementary Table 3). Even after adjusting for age, TMEM106B levels were positively correlated with protein sets whose gene expression increases with age in the human frontal cortex [13] and mouse neocortex [50], and negatively correlated with proteins sets whose gene expression decreases in the ageing human frontal cortex [13] and mouse hypothalamus [51] (Fig. 3C). Interestingly, TMEM106B levels were also positively associated with proteins whose gene expression is increased in the hippocampus during incipient AD [52].

### Myelin lipid content is decreased in carriers of the rs1990622-A dementia risk allele

**Figure 4:**
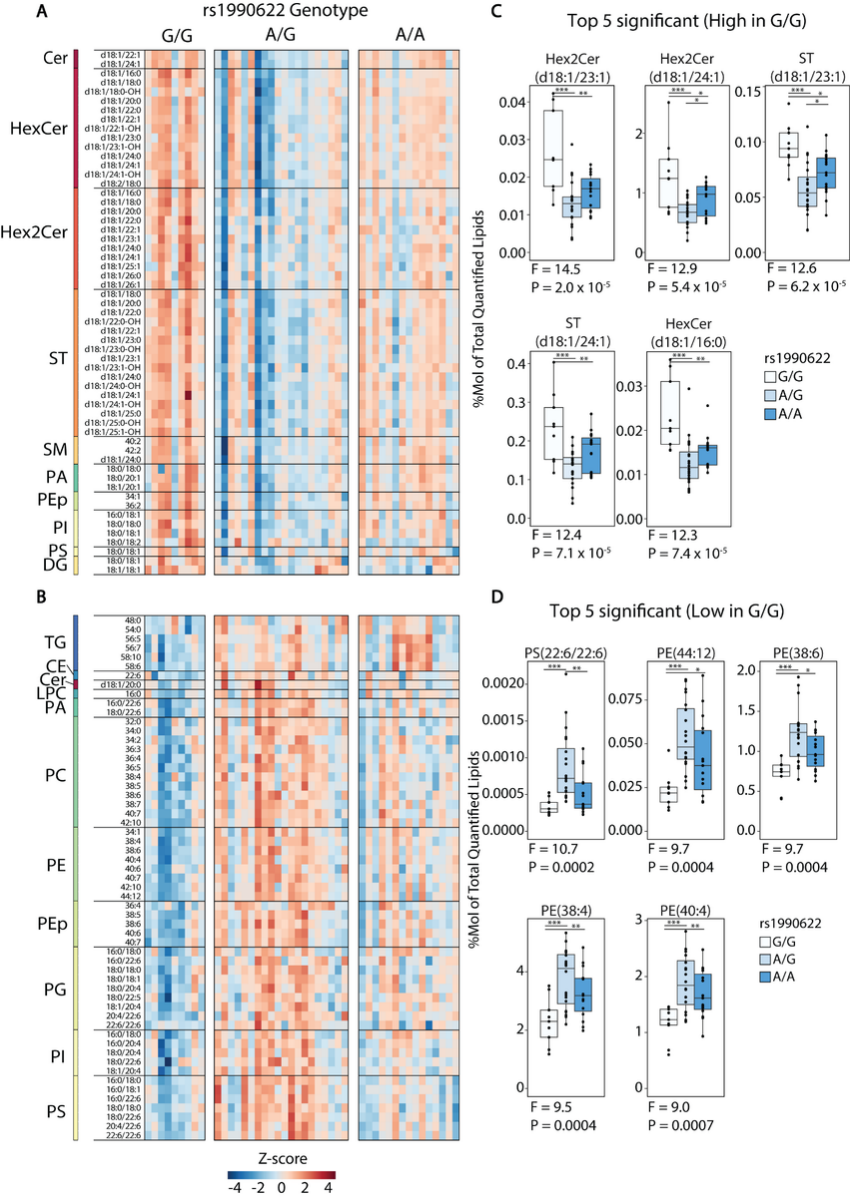
*TMEM106B* rs1990622 genotype affects the hippocampal lipidome. (A-B) Heatmap of lipids that were significantly affected by rs1990622 genotype (N_G/G_=9, N_A/G_=20, N_A/A_=15), in individuals with *APOE e3/e3* genotype. Each row represents a lipid that was significantly more abundant (A) or less abundant (B) in rs1990622-G/G individuals by ANOVA at Q<0.05. (C-D) Top five lipid species that are more abundant (C) and less abundant (D) in rs1990622-G/G individuals ranked by P value. Lipids values are expressed as a molar % of total lipid. Cer: ceramide; DG: diacylglycerol; HexCer: hexosylceramide; Hex2Cer: dihexosylceramide; LPA: lysophosphatidic acid; LPC: lysophosphatidylcholine; PA: phosphatidic acid; PC: phosphatidylcholine; PE: phosphatidylethanolamine; PEp: phosphatidylethanolamine plasmalogen; PG: phosphatidylglycerol; PI: phosphatidylinositol; PS: phosphatidylserine; SM: sphingomyelin; ST: sulfatide; TG: triglyceride; CE: cholesterol ester.

Given that (i) many of the most important gene variants affecting dementia risk regulate lipid transport and catabolism [53], (ii) structural modelling has proposed a lipid binding function for TMEM106B [54], and (iii) TMEM106B protein levels were correlated with proteins regulating fatty acid and lipid metabolism, we used lipidomic analysis to determine how dementia risk allele rs1990622-A affects lipid composition in the hippocampus of cognitively normal humans. A total of 234 phospholipids, sphingolipids, and neutral lipids were quantified by LC-MS/MS. As variants in *APOE*, which encodes a lipid transport protein, are the most significant determinant of genetic risk for dementia [53], we first determined whether lipid levels were significantly affected by *APOE* genotype. In one-way ANOVA comparing 46 cases with a risk-neutral *APOE* e3/e3 genotype, 12 with a protective e3/e2 or e2/e2 genotype (e2 carriers), and 12 with a risk-increasing e3/e4 genotype, no lipids were significantly regulated (Q < 0.05) at the level of individual lipid species or lipid class totals (Supplementary Tables 4 and 5). Similarly, no lipids were regulated by rs1990622 genotype at Q < 0.05 in the full sample set (Supplementary Table 6). However, when the effect of rs1990622 was tested in *APOE* e3/e3 samples alone, 57 lipids were more abundant and 57 less abundant in carriers of the protective rs1990622-G/G genotype compared to rs1990622-A allele carriers (Fig. 4A–D, and Supplementary Table 7).

**Figure 5:**
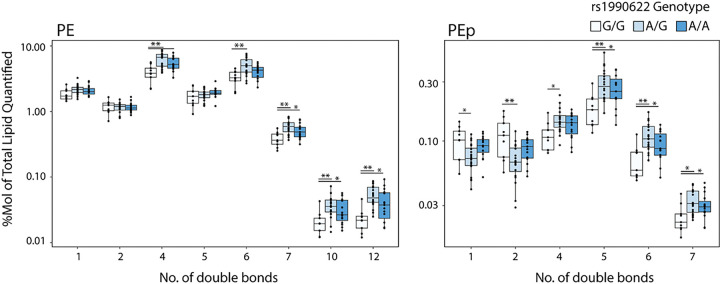
Rs1990622 affects the degree of unsaturation in phosphatidylethanolamine (PE) and PE plasmalogen (PEp). PE and PEp levels as a function of the number of double bonds in the acyl chains, grouped by rs1990622 genotype (*APOE* e3/e3 genotype only; N_G/G_=9, N_A/G_=20, N_A/A_=15). Statistical significance was determined by ANOVA with Tukey’s post-hoc test: *P<0.05, **P<0.01, ***P<0.001.

Of the 57 lipids that were higher in G/G individuals, 45 were sphingolipids, specifically hexosylceramides (HexCer), dihexosylceramides (Hex2Cer), sulfatides (ST), sphingomyelins (SM), and ceramides (Cer). Over 99% of HexCer in the brain is galactosylceramide [55], which makes up 20–25% of myelin lipids [20]. Together with ST (4–5% of myelin lipid), galactosylceramide is essential for myelin stability, and these lipid classes are unique to myelin in the CNS [56]. All but two of the lipids that were lower in rs1990622-G/G genotype individuals consisted of phospholipids and triglycerides (TG), particularly those with polyunsaturated fatty acid chains (Fig. 4B and D). The effect of rs1990622 on saturated versus polyunsaturated lipids was particularly apparent for phosphatidylethanolamine plasmalogens (PEp): PEp species with 1 or 2 double bonds were higher in people with a rs1990622-G/G genotype, whereas those with 4 or more double bonds were decreased (Fig. 5).

**Figure 6:**
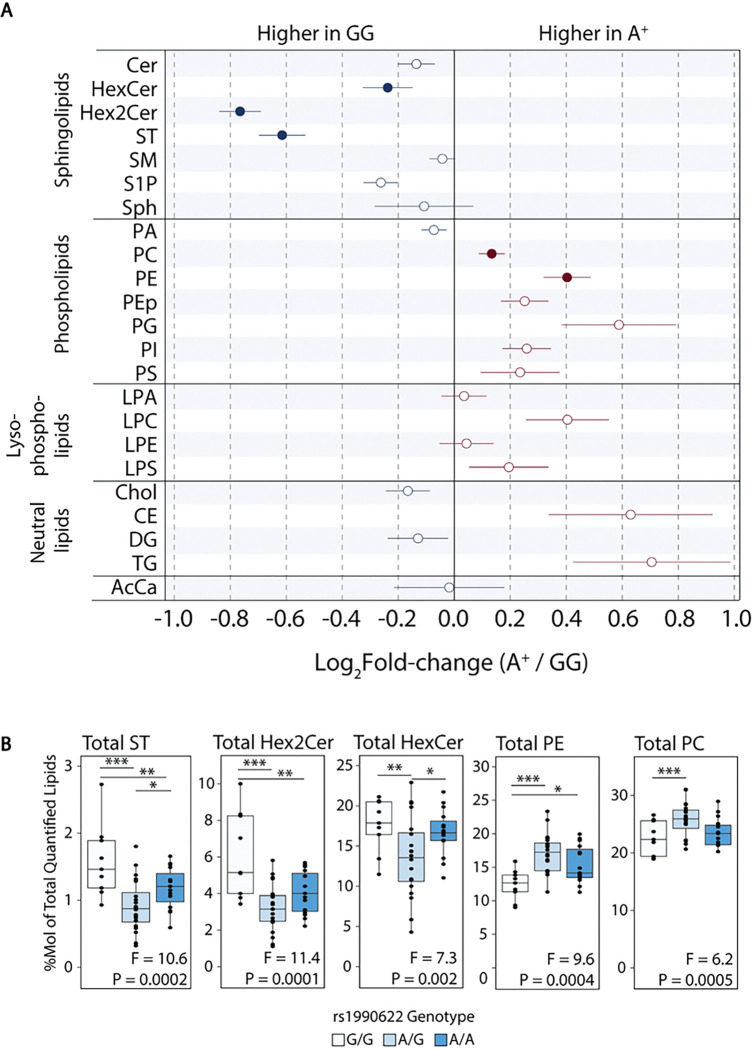
Higher ST and Hex2Cer, and lower PE, in carriers of the protective rs1990622-G/G genotype. (A) Summary of changes in lipid class totals in rs1990622-A allele carriers (denoted as A^+^) relative to rs1990622-G/G homozygotes (*APOE* e3/e3 genotype only). Filled circles indicate lipid classes that differed significantly between the three rs1990622 genotypes (G/G, A/G, and A/A) by ANOVA, at Q<0.05 (*APOE* e3/e3 genotype only; N_G/G_=9, N_A/G_=20, N_A/A_=15). (B) Lipid class totals as a function of rs1990622 genotype for the five lipids significant by ANOVA. F and P values are shown at the bottom of each plot and results of Tukey’s post-hoc comparisons are shown above (* P<0.05, ** P<0.01, *** P<0.001). Cer: ceramide; HexCer: hexosylceramide; Hex2Cer: dihexosylceramide; ST: sulfatide; SM: sphingomyelin; S1P: sphingosine 1-phosphate; Sph: sphingosine; PA: phosphatidic acid; PC: phosphatidylcholine; PE: phosphatidylethanolamine; PEp: phosphatidylethanolamine plasmalogen; PG: phosphatidylglycerol; PI: phosphatidylinositol; PS: phosphatidylserine; LPA: lysophosphatidic acid; LPC: lysophosphatidylcholine; LPE: lysophosphatidylethanolamine; LPS: lysophosphatidylserine; Chol: cholesterol; CE: cholesteryl ester; DG: diacylglycerol; TG: triacylglycerol; AcCa: acylcarnitine.

At the lipid class level (sum of individual lipid species), Hex2Cer and ST were higher, and PE was lower, in the rs1990622-G/G compared to both A/G and A/A genotype groups (Fig. 6A and B, Supplementary Table 8). Total HexCer was higher, and PC was lower, in the G/G compared to the A/G genotype group, but these lipid class totals did not differ between the G/G and A/A genotype groups. Other sphingolipid, phospholipid, and neutral lipid class totals were unaffected by rs1990622 genotype at Q < 0.05. Despite the associations between lipids and rs1990622, TMEM106B protein levels were not significantly correlated with any lipids or lipid class totals, and no individual lipids or lipid class totals were significantly correlated with age (Supplementary Tables 9 and 10).

## Discussion

The risk of dementia doubles every five years after age 65 [1], thought to result from age-dependent changes that sensitize the brain to neurodegeneration. Employing a large set of post-mortem samples, we have identified proteins whose abundance is significantly affected by physiological ageing in the hippocampus of humans aged 65 and older. Ribosomal proteins, respiratory complex proteins, and proteins involved in amino acid and lipid metabolism were increased with age, while half of the significantly decreased proteins were regulators of the cytoskeleton and cell adhesion. Lysosomal protein TMEM106B was increased with the greatest effect size, and this was specific to carriers of the rs1990622-A dementia risk allele. While *APOE* genotype had no significant effect on hippocampal lipids in our dataset, rs1990622-A was associated with lower myelin sphingolipid and higher polyunsaturated phospholipid content in carriers of the dementia risk-neutral *APOE* e3/e3 genotype, providing the first direct evidence for an effect of this dementia risk allele on brain lipids. Increased TMEM106B protein levels with ageing and/or altered myelin lipid homeostasis are likely contributors to the increased susceptibility of rs1990622-A allele carriers to neurodegeneration.

The majority of proteins whose levels were significantly correlated with age were involved in constitutive metabolic and cellular processes such as lipid and amino acid metabolism, translation, and control of cell adhesion and the cytoskeleton. Increased levels of the lysosomal proteins TMEM106B and ASAH1 (acid ceramidase) concur with prior studies demonstrating increased abundance of lysosomal proteins with ageing in human brains [14, 18], as well as increased lifetimes for lysosomal proteins in old compared to young adult mice [10]. A recent study of frontal cortex proteins affected by ageing and AD [18] did not report data on TMEM106B but showed the same highly significant positive and negative correlations with age for ASAH1 and CORO1A, respectively. Our observation of a general increase in electron transport chain proteins with age is in agreement with published studies on the rat hippocampus [16], but contrasts with other studies reporting either no significant change in mitochondrial proteins [18] or decreased levels of electron transport chain proteins in the hippocampus of humans aged > 90 years, compared to those aged 20 to 49 years [14, 17]. Similarly, while our study demonstrated an overall increase in abundance for ribosomal protein subunits with increasing age, prior studies have reported dissonant findings regarding changes to ribosomal protein abundance across humans, mice, monkeys, and fish [11, 15, 18, 57]. These differences are most likely attributed to experimental design. Rather than comparing mean protein levels between young and old age groups, as has been done in prior studies, we used a large sample size of individuals over the age of 65 and identified proteins whose abundance correlated with age. This allowed us to identify age-dependent changes to the proteome across the age range that is most relevant to the onset of dementia. We also note that sample sizes in prior studies with human hippocampus were limited to 3 or 4 samples per group [14, 17]. An important limitation of our study is that overrepresentation of ribosomal and electron transport chain subunits may reflect the natural bias of proteomics towards more abundant proteins. Nonetheless, our findings are supported by the substantial overlap between our proteomic dataset and published gene expression data on human frontal cortex [13], in terms of proteins that increased or decreased with age.

Higher hippocampal TMEM106B protein levels as a function of age at death could indicate either that TMEM106B levels increase with ageing, or that people with higher hippocampal TMEM106B live longer. The former is much more likely, given that increased TMEM106B with ageing was seen only in people with the rs1990622-A dementia risk allele. This association also implies that higher TMEM106B levels with ageing are detrimental to the hippocampus. Rs1990622-A is relatively unique among genetic risk factors for dementia, as it increases risk for all major forms of dementia [39]. This SNP is also associated with lower cognitive scores in adults without neurodegenerative diseases [59, 60] and a transcriptomic signature that characterizes accelerated ageing in people > 65 years of age [60]. While rs1990622-A has previously been associated with higher *TMEM106B* mRNA levels in the frontal cortex of people with FTD [42] and hippocampus of healthy donors [40], this is the first study to demonstrate that rs1990622-A increases TMEM106B protein levels with physiological ageing. Rs1990622 is in perfect linkage disequilibrium with another non-coding SNP in the *TMEM106B* gene, rs1990620 [40], which affects recruitment of the chromatin-organising protein CCCTC-binding factor (CTCF), leading to increased *TMEM106B* gene expression [40]. This mechanism may contribute to the age-dependent increase of TMEM106B protein in rs1990622-A carriers.

TMEM106B protein levels were correlated with lipid metabolic and myelination protein networks that are upregulated in response to demyelination, AD, and brain ageing [52, 61]. QKI, which was correlated with TMEM106B at Q < 0.05, is a master regulator of oligodendrocyte fatty acid metabolism and myelin maintenance [47]. This protein is also necessary for the microglial phagocytosis of myelin debris [62], which may explain its association with both brain ageing and incipient AD (Fig. 3C). TMEM106B-associated protein PLXNB1 is also associated with brain ageing and severity of AD pathology [63] and has been proposed as a key driver of AD in the ageing brain [64]. TMEM106B may therefore form part of a network of proteins whose levels are increased in the ageing brain and incipient AD.

The ubiquity of TMEM106B amyloid fibrils across a broad range of neurodegenerative diseases [33–35], and the presence of these fibrils in older neurologically normal individuals but not young brains [33], implicates these fibrils in the neurodegenerative process. The age-dependent elevation of TMEM106B levels in rs1990622-A risk allele carriers could contribute to the formation of these amyloid fibrils, given that protein levels above a critical concentration are prerequisite for fibrilization and amyloid formation [65].

Higher myelin sphingolipid (particularly ST and Hex2Cer) content in rs1990622-G/G individuals could result from higher overall myelin content or a more specific effect of this SNP on lipid metabolism or transport. The latter is supported by the observation that rs1990622 significantly affected the hippocampal lipidome but not the proteome (including myelin proteins), however this should be interpreted with caution since the proteomic results were subject to more stringent FDR correction (2091 proteins versus 246 lipids). Myelin is also enriched in PEp [56], and rs1990622-G/G was associated with higher levels of saturated, and lower levels of polyunsaturated, PEp. As a stable membrane with low fluidity, myelin contains more saturated/monounsaturated and less polyunsaturated lipids than other cell membranes [56]. In contrast, vesicular membranes often contain more polyunsaturated phospholipids that facilitate membrane fusion [66]. The protective rs1990622-G/G genotype is therefore associated with higher myelin lipid content. The effect of rs1990622 on hippocampal lipids may be distinct from TMEM106B protein levels since we observed no correlation between TMEM106B levels and myelin sphingolipids. However, the expression of genes involved in galactosylceramide biosynthesis is decreased in TMEM106B knockout mice [38], suggesting that TMEM106B regulates sphingolipid metabolism in oligodendrocytes, the only cells in the nervous system that synthesize galactosphingolipids [56]. We note that rs1990622 is in perfect linkage disequilibrium with the TMEM106B coding variant rs3173615 (T185S) [41], which is known to affect the association of TMEM106B with at least one other protein (CHMP2B) in endosome sorting complexes [67]. It is therefore possible that the effect of rs1990622 on myelin lipid content is attributed to a subtle change in the protein’s structure and function resulting from the linked T185S variant.

Age-dependent myelin loss is increasingly regarded as a probable sensitizing factor for cognitive decline and dementia [68–70]. We speculate that the rs1990622-G/G genotype could protect against cognitive decline and dementia by preserving myelin integrity with ageing. Rs1990622-A synergizes with heterozygous *GRN* loss of function mutations to increase the risk for FTD [71]. It would therefore be of interest to determine if these rare *GRN* mutations are also associated with lower myelin sphingolipid content, and how this is affected by rs1990622-A.

Although the large sample size of neurologically-normal brain donors empowered us to probe the effects of age, *APOE*, and *TMEM106B* genotype on hippocampal protein and lipid composition in individuals aged over 65, our study was limited to nine individuals with both *APOE* 3/ 3 and rs1990622-G/G genotype. Our demonstration that rs1990622 genotype affects lipid metabolism should therefore be validated in another sample cohort, ideally sampling a white matter region given the impact of rs1990622 on myelin lipids. Rs1990622-A interacts with *APOE* genotype to increase risk for AD in the Han Chinese population [72]. The current study was underpowered to test the combined effects of *APOE* genotype and rs1990622 on the hippocampal lipidome.

## Conclusions

In summary, this study establishes that TMEM106B protein levels increase with age over 65, as part of a network of proteins that increase with age in the human hippocampus. Increased hippocampal TMEM106B with ageing is driven by the rs1990622-A dementia risk allele in the *TMEM106B* gene, and we provide the first experimental evidence that rs1990622-A regulates brain lipid metabolism, placing it among the growing list of genetic risk factors for dementia involved in lipid homeostasis. Lastly, the matched proteomic and lipidomic datasets generated in this project should prove a valuable resource for neuroscience, ageing, and dementia research.

## Data Availability

The proteomic and lipidomic datasets supporting the conclusions of this article are provided in Additional File 1. The proteomic data is also deposited with ProteomeXchange, and Lipidomic data with Metabolomics Workbench.

## List Of Abbreviations

AD: Alzheimer’s disease
NFT: Neurofibrillary tangle
HS-ageing: Hippocampal sclerosis with ageing
TDP-43: TAR DNA binding protein 43
LATE: Limbic-predominant age-related TDP-43 encephalopathy
FTD: Frontotemporal dementia
DLB: Dementia with Lewy bodies
TMEM106B: Transmembrane protein 106B
TCEP: tris(2-carboxyethyl)phosphine
LC-MS/MS: Liquid chromatography-tandem mass spectrometry
SNP: Single-nucleotide polymorphism
BUME: Butanol-methanol
FDR: False discovery rate
GSEA: Gene set enrichment analysis
ERLIN2: ER lipid raft associated 2
DHRS7: Dehydrogenase/reductase 7
QKI: Protein quaking I
PLXNB1: Plexin B1
LMNA: Lamin A/C
HexCer: Hexosylceramide
Hex2Cer: Dihexosylceramide
ST: Sulfatide
SM: Sphingomyelin
Cer: Ceramide
TG: Triglyceride
PEp: Phosphatidylethanolamine plasmalogen
PE: Phosphatidylethanolamine
ASAH1: Acid ceramidase 1
CTCF: CCCTC-binding factor
